# Draft Genome Sequences of 12 Histophilus somni Strains Isolated from Feedlot Cattle in Alberta, Canada

**DOI:** 10.1128/MRA.00864-19

**Published:** 2019-11-14

**Authors:** Mohammad Mostafa Nazari, Krishna Bhatt, Neil Rawlyk, Andrew A. Potter, Karen Liljebjelke

**Affiliations:** a Faculty of Veterinary Medicine, Department of Ecosystem and Public Health, University of Calgary, Calgary, Alberta, Canada; b Vaccine and Infectious Disease Organization–International Vaccine Centre, University of Saskatchewan, Saskatoon, Saskatchewan, Canada; University of Maryland School of Medicine

## Abstract

Histophilus somni is a Gram-negative opportunistic pathogen associated with respiratory disease in cattle. Here, we report the draft genome sequences of 12 Histophilus somni strains isolated from feedlot cattle in Alberta, Canada, which were diagnosed with respiratory disease.

## ANNOUNCEMENT

Histophilus somni, formerly Haemophilus somnus, is a member of the family *Pasteurellaceae*. Histophilus somni, along with two other members of *Pasteurellaceae* (Pasteurella multocida and Mannheimia haemolytica), is associated with bovine respiratory disease (BRD) complex (1). An opportunistic pathogen, H. somni is also responsible for a multisystemic disease called histophilosis in cattle, sheep, and goats (2–4). A better understanding of the genome of H. somni, including antimicrobial resistance and horizontal gene transfer, is needed to understand BRD in feedlot cattle.

The raw FASTQ files of 12 H. somni whole-genome sequences were obtained from the Vaccine and Infectious Disease Organization–International Vaccine Centre (VIDO-InterVac), Saskatoon, Canada. These short-read whole genomes were previously aligned against a reference genome for the detection of single nucleotide polymorphisms and insertion and deletion sites (5), as well as for prediction of protein-coding regions (6). For this work, the genomes were assembled *de novo* and annotated using the methods described below, and this information was deposited in NCBI GenBank.

These genome sequences originate from six H. somni strains isolated from the heart and lung tissue and synovial fluid of feedlot calves between 2012 and 2013 and from six H. somni strains isolated from feedlot cattle lung tissue in the 1980s (5).

Amies transport medium with charcoal was used to collect and transport samples (5). Isolates were cultured on tryptic soy agar with 5% sheep blood and incubated for 36 to 48 h at 37°C in a 5% CO_2_ atmosphere (5–7). Genomic DNA was extracted with a Genomic-tip (Qiagen, Toronto, Ontario, Canada), as previously described (5). Genomic DNA libraries were prepared using a Nextera XT library prep kit (Illumina, San Diego, CA, USA), and whole-genome sequencing was done using the Illumina MiSeq 500 platform, with a paired-end 2 × 150-bp read type, as previously described (5).

FastQC v1.0.0 (https://www.bioinformatics.babraham.ac.uk/projects/fastqc) and QUAST v5.0.2 (http://quast.sourceforge.net/quast) were used for quality assessment and trimming of sequenced files before and after assembly, respectively (8, 9). Default parameters were used for all *in silico* software, unless otherwise specified. Quality control and DNA trimming of the raw FASTQ files included removing low-quality base pairs at each read terminal (quality [Q] score, <20), as well as reads of less than 50 nucleotide base pairs, that were >10% Ns, and those with a >15-bp overlapping sequence with the Illumina adaptors. FASTQ files with a Q score of at least 98% were used for further *in silico* analysis. The genomes were assembled *de novo* using SPAdes v3.13.0 (http://cab.spbu.ru/software/spades/) (10). The final annotation of sequences was performed using the NCBI Prokaryotic Genome Annotation Pipeline (PGAP) (https://www.ncbi.nlm.nih.gov/genome/annotation_prok/) with default thresholds (11).

### Data availability.

The genome sequences and associated data for 12 H. somni strains were deposited in NCBI GenBank, the European Nucleotide Archive (ENA), and the DNA Data Bank of Japan (DDBJ), under the accession numbers provided in Table 1.

**TABLE 1:** H. somni genome characteristics and accession numbers

Isolate	NCBI accession no.				ENA accession no.	DDBJ accession no.	G+C content (%)	Avg length of raw reads (bp)	Total no. of reads	Genome size (bp)	Total no. of contigs	No. of rRNAs	No. of tRNAs	No. of ncRNAs^*a*^	Total no. of genes
	GenBank	BioSample	BioProject	SRA											
KLM-01	NZ_SKBV00000000	SAMN11042644	PRJNA525156	SRR9761912	SRP216147	SRX6518714	36.9	150	387,096,900	2,361,113	199	5	48	4	2,283
KLM-03	NZ_SMNW00000000	SAMN11094176	PRJNA526281	SRR9761918	SRP216148	SRX6532989	37.3	150	459,494,700	2,229,261	132	4	44	4	2,078
KLM-04	NZ_SNRV00000000	SAMN11132143	PRJNA527266	SRR9776588	SRP216160	SRX6539641	37.4	150	408,072,300	2,271,789	178	4	44	4	2,151
KLM-06	NZ_SOYZ00000000	SAMN11191546	PRJNA528478	SRR9824922	SRP216238	SRX6581597	36.9	150	411,876,300	2,363,734	199	5	48	4	2,286
KLM-07	NZ_SPKI00000000	SAMN11264820	PRJNA529326	SRR9824930	SRP216239	SRX6581605	37.1	150	457,396,500	2,092,744	97	4	44	4	1,909
KLM-08	NZ_SSCM00000000	SAMN11360228	PRJNA531494	SRR9824931	SRP216240	SRX6581606	37.2	150	420,674,400	2,149,571	127	7	44	4	1,990
KLM-09	NZ_SSCN00000000	SAMN11360446	PRJNA531503	SRR9824960	SRP216242	SRX6581635	37.1	150	296,814,900	2,079,435	97	4	42	4	1,900
KLM-10	NZ_SSCQ00000000	SAMN11360900	PRJNA531509	SRR9825144	SRP216251	SRX6581813	36.7	150	413,164,800	2,233,495	147	4	46	4	2,119
KLM-11	NZ_SSCO00000000	SAMN11360902	PRJNA531510	SRR9825155	SRP216252	SRX6581824	37.1	150	3741,13,800	2,090,388	106	4	44	4	1,906
KLM-12	NZ_SSCP00000000	SAMN11360904	PRJNA531512	SRR9825483	SRP216254	SRX6582152	37.2	150	435,419,400	2,128,480	129	4	45	4	1,974
KLM-13	NZ_SSCR00000000	SAMN11372139	PRJNA531670	SRR9825531	SRP216256	SRX6582199	37.0	150	429,169,200	2,406,339	197	4	47	4	2,339
KLM-14	NZ_SUKB00000000	SAMN11475348	PRJNA534002	SRR9825577	SRP216257	SRX6582245	37.2	150	424,964,700	2,228,888	128	4	44	4	2,073

a ncRNAs, noncoding RNAs.
